# Quality of life in primary sclerosing cholangitis: a systematic review

**DOI:** 10.1186/s12955-021-01739-3

**Published:** 2021-03-20

**Authors:** Elena Marcus, Paddy Stone, Anna-Maria Krooupa, Douglas Thorburn, Bella Vivat

**Affiliations:** 1 Marie Curie Palliative Care Research Department, Division of Psychiatry, University College London, 6th Floor, Maple House, 149 Tottenham Court Road, London, W1T 7NF UK; 2 University College London Institute of Liver and Digestive Health, UCL Royal Free Campus, Royal Free Hospital, London, UK; 3 Sheila Sherlock Liver Unit, Royal Free Hospital, London, UK

**Keywords:** Primary sclerosing cholangitis, Quality of life, Questionnaires

## Abstract

**Background:**

Primary sclerosing cholangitis (PSC) is a rare bile duct and liver disease which can considerably impact quality of life (QoL). As part of a project developing a measure of QoL for people with PSC, we conducted a systematic review with four review questions. The first of these questions overlaps with a recently published systematic review, so this paper reports on the last three of our initial four questions: (A) How does QoL in PSC compare with other groups?, (B) Which attributes/factors are associated with impaired QoL in PSC?, (C) Which interventions are effective in improving QoL in people with PSC?.

**Methods:**

We systematically searched five databases from inception to 1 November 2020 and assessed the methodological quality of included studies using standard checklists.

**Results:**

We identified 28 studies: 17 for (A), ten for (B), and nine for (C). Limited evidence was found for all review questions, with few studies included in each comparison, and small sample sizes. The limited evidence available indicated poorer QoL for people with PSC compared with healthy controls, but findings were mixed for comparisons with the general population. QoL outcomes in PSC were comparable to other chronic conditions. Itch, pain, jaundice, severity of inflammatory bowel disease, liver cirrhosis, and large-duct PSC were all associated with impaired QoL. No associations were found between QoL and PSC severity measured with surrogate markers of disease progression or one of three prognostic scoring systems. No interventions were found to improve QoL outcomes.

**Conclusion:**

The limited findings from included studies suggest that markers of disease progression used in clinical trials may not reflect the experiences of people with PSC. This highlights the importance for clinical research studies to assess QoL alongside clinical and laboratory-based outcomes. A valid and responsive PSC-specific measure of QoL, to adequately capture all issues of importance to people with PSC, would therefore be helpful for clinical research studies.

**Supplementary Information:**

The online version contains supplementary material available at 10.1186/s12955-021-01739-3.

## Background

Primary sclerosing cholangitis (PSC) is a rare and chronic cholestatic liver disease, characterised by inflammation and fibrosis of the bile ducts [1]. Over time PSC can lead to liver cirrhosis, in some cases progressing to liver failure [2]. Approximately 70% of people with PSC also have a concurrent diagnosis of inflammatory bowel disease (IBD) [3] and there is an increased risk of hepatobiliary cancers and colorectal cancer [4, 5]. Currently no treatment is available to cure PSC, or slow disease progression, and liver transplantation is the only intervention known to extend survival [6]. Although the condition is rare, 10–15% of liver transplants in Europe are performed for PSC [7], and it is the leading indication for liver transplant among autoimmune liver conditions in the UK and the US [8]. Early in the disease, symptoms tend to be rare and approximately 40–50% of people are asymptomatic at diagnosis [6, 9]. However, with limited treatment options, people with PSC can live for many years with a number of debilitating symptoms such as fatigue, itch, and pain, as well as the emotional burden of an uncertain future [6, 10], all of which can impact on quality of life (QoL) [11, 12].

In 2019 PSC was identified as a top 10 research priority in non-alcohol related liver and gallbladder disorders in the UK [13], and the lack of treatments for PSC indicated as a major concern. Defining endpoints for clinical trials in PSC, however, has its challenges due to the unpredictable and prolonged clinical course of the condition [14]. Patient-reported outcome measures (PROMs), including assessments of QoL, are important for use in clinical trials, addressing health-related experiences from the patient perspective [15]. The assessment of patients’ experiences in clinical research is particularly warranted for chronic conditions, such as PSC, which can have a long-term impact on functioning and well-being [16, 17]. Capturing these experiences in a patient-centred way is necessary to enable holistic assessment of the safety and efficacy of new interventions for people with PSC [18].

As the first stage in a doctoral project developing a measure of QoL for people with PSC in the UK, we searched the literature for what is known about QoL in this population [19]. That search only found few existing reviews, all of which focused narrowly on QoL in PSC. One of these, a Cochrane review of pharmacological interventions for PSC, included QoL as an outcome measure [20]; another explored the impact of itch on QoL for cholestatic liver disease [21]. There was therefore a lack of clarity about how QoL had been measured in this population, which factors were important determinants of QoL in PSC, and how the condition affected QoL. Four more reviews by other groups have since been published, and were identified in later updates to the initial search: a systematic review assessing PROMs used in PSC [22], a systematic review identifying PRO instruments and concepts used in the PSC literature [23], a literature review exploring QoL in cholestatic disease [24], and a scoping review exploring the impact of PSC on psychological well-being [25].

The aim of this part of the doctoral study was to systematically review QoL-related outcomes in PSC. Due to the paucity of published literature, we formulated four separate review questions: (1) ‘Which validated questionnaires have been used to assess QoL in people with PSC?’, (2) ‘How does QoL in people with PSC compare with QoL in other groups?’, (3) ‘What factors are associated with impaired QoL in people with PSC?’, and (4) ‘Which interventions are effective in improving QoL in people with PSC?’. A separate systematic review [22] covered similar ground to our Review question [1], but was a broader review, identifying all PROMs used in PSC, and critically appraising included measures. Our review retrieved only validated measures of QoL, so is less broad and does not add to the work conducted by this other group. Our paper therefore reports only the findings from the last three of our review questions, identified as (A), (B) and (C) in the sections below.

## Methods

This review was conducted and reported in line with the guidelines of the Preferred Reporting Items for Systematic Reviews and Meta-Analyses (PRISMA). The review was registered with the International Prospective Register of Systematic Reviews (PROSPERO), registration number: CRD42017071729 [26].

### Search strategy

We developed a single systematic search strategy to locate relevant evidence across all the review questions: (A) How does QoL in people with PSC compare with QoL in other groups? (B) What factors are associated with impaired QoL in people with PSC? and (C) Which interventions are effective in improving QoL in people with PSC? We searched Embase, MEDLINE, PsycINFO, SCOPUS, and Web of Science databases from inception to 4 June 2019, with subsequent updates on 31 March 2020 [19], and then 1 November 2020. The key search terms used were ‘primary sclerosing cholangitis’ and ‘quality of life’, along with synonyms and related terms. The search strategy was initially developed for MEDLINE (see the Additional file 1 for the full MEDLINE search strategy), and then translated for use in the other databases. In addition to the electronic database searches, we hand-searched the reference lists of included studies and relevant systematic reviews and conducted a forward citation search for included studies in Google Scholar. Authors were contacted where key data were missing from the published report. Authors of identified conference abstracts were contacted for full-text papers.

### Inclusion and exclusion criteria

Studies were eligible for inclusion if they were primary studies of adult participants with PSC (≥ 18 years) which assessed QoL and were published from inception to the final database search (Nov 2020). For the purpose of this review we drew on work exploring QoL in relation to cancer, that is, health-related QoL [27], and defined QoL as a multi-dimensional construct comprising the impact of illness or treatment on a person’s functioning and well-being in physical, psychological and social domains. Due to the paucity of literature exploring QoL in PSC, we included studies that used multi-dimensional QoL questionnaires, as well as studies that used questionnaires which focused on specific domains of QoL: physical symptoms (e.g. gastro-intestinal symptoms), psychological well-being and social functioning. When describing findings from multi-dimensional QoL questionnaires, we use the term *QoL*. Where studies report specific domains of QoL (e.g. depression), we explicitly name these. To include studies where participants had a range of conditions, we required at least 50% of the study sample to have PSC. Where < 50% of the sample had PSC, authors were contacted to request disaggregated data. We excluded studies of children and adolescents with PSC. Non-primary studies, such as systematic reviews and editorials, were also excluded. Due to resource constraints we limited publications to English language papers.

Specific inclusion criteria for each review question were as follows. Question A: Any primary study, including cohort, cross-sectional and case–control studies, comparing QoL outcomes between PSC participants and any other group. Question B: Any primary study, including cohort, cross-sectional and case–control studies, exploring the association of any factor or attribute with QoL. Question C*:* Any randomised or non-randomised controlled study comparing any intervention with any comparator. Before-and-after studies were excluded.

### Selection of studies

We exported citations from each electronic database search to Endnote (version X7) and removed duplicates. We screened titles and abstracts of identified studies for inclusion against agreed criteria. Two reviewers (EM, AMK) independently screened 10% of references, and, because the inter-rater reliability was good (88% agreement), one reviewer screened the remaining references. All primary-level studies included after the first scan of citations were acquired in full and re-evaluated for eligibility. Two reviewers (EM, AMK) independently screened all full-text papers using the inclusion criteria for reference. The percentage agreement was good (81%), and after discussion all disagreements were resolved.

### Data extraction

A single reviewer (EM) extracted the following data using a pre-defined form: study design, date of publication, country of origin, setting, sample size, participant inclusion/exclusion criteria, participant characteristics, name of utilised QoL tool(s), comparator groups (for Question A), factors or attributes correlated with QoL (for Question B), and type/dose of intervention and comparator (for Question C). The following outcome data were extracted where relevant and available: type of outcome, name of outcome measure, direction of scale, mean, standard deviation, number of events, effect size, confidence intervals, *p* values, and correlation/regression coefficients (for Question B). Where these data were not reported, we extracted narrative descriptions of findings from the published report.

### Quality appraisal

We assessed risk of bias at the study level using standard checklists. Different checklists were used depending on the design of the study. We assessed randomised controlled trials (RCTs) with Version 1 of the Cochrane Collaboration’s Questionnaire for Assessing Risk of Bias in Randomised Trials [28], cohort and case–control studies with The Newcastle–Ottawa Scale (NOS) [29], and cross-sectional studies and surveys with the Critical Appraisal Questionnaire to Assess the Quality of Cross-sectional Studies (AXIS) [30]. To aid the comparison of quality across studies, we assigned each observational study a quality rating following criteria published by Harbour and Miller [31] (Table 1). We did not assign individual study quality ratings to the RCT evidence as this is not recommended [28] and only few RCTs were included (n = 8).

**Table 1:** Quality rating for individual studies

Quality rating	Criteria
High	All or most of the checklist criteria have been fulfilled, where they have not been fulfilled the conclusions are very unlikely to alter
Moderate	Some of the checklist criteria have been fulfilled, where they have not been fulfilled, or not adequately described, the conclusions are unlikely to alter
Low	Few or no checklist criteria have been fulfilled and the conclusions are likely or very likely to alter

### Data analysis

We synthesised data narratively. Combining data in meta-analyses was inappropriate due to differences in outcome measures, participant groups and interventions. In addition, many studies did not report data in a format suitable for meta-analysis (e.g. reporting findings narratively). We report effect sizes, confidence intervals, and/or *p* values if these were available in the original reports. To explore heterogeneity of findings for Question A, we conducted two sensitivity analyses *post-hoc*. The first limited the evidence to studies with a lower risk of bias (rated as moderate or high quality). The second limited the evidence to studies which age- and gender-matched PSC participants to the comparator group.

## Results

### Study selection

**Fig. 1:**
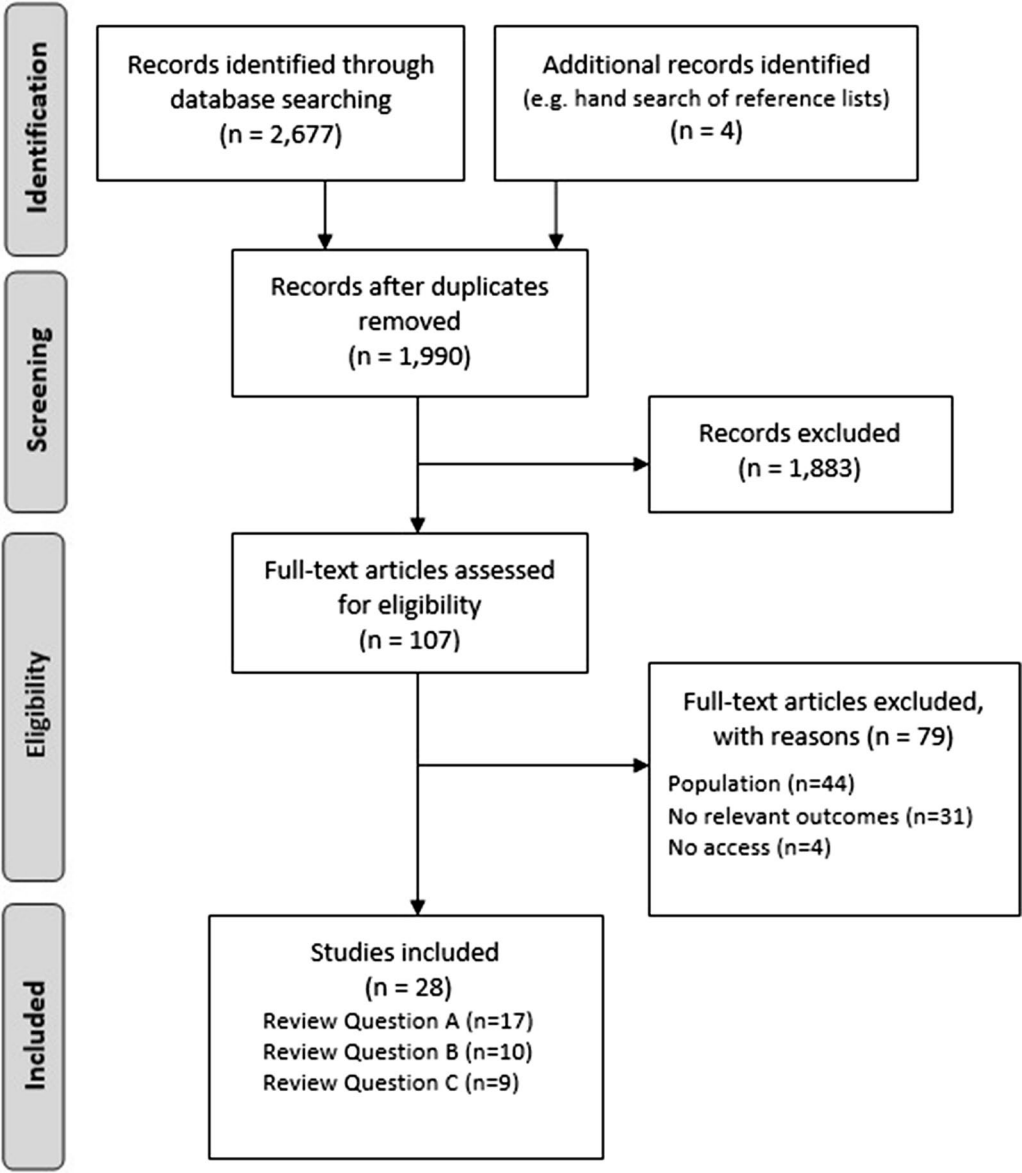
PRISMA flow diagram

We identified 2677 records, 1990 after the removal of duplicates, and 107 after screening titles and abstracts (Fig. 1). Four additional articles were identified through a hand search of reference lists of relevant systematic reviews and included studies. Following a full-text appraisal, 28 studies (reported across 29 individual manuscripts) were included across the three review questions; two papers reported on the same data set [32, 33].

### Question A: How does QoL in people with PSC compare with QoL in other groups?

#### Study characteristics

Seventeen studies met the inclusion criteria: 13 cross-sectional studies, two case–control studies and two cohort studies (Table 2). Each study compared QoL outcomes between PSC participants and up to seven other comparator groups. These comprised: (1) control groups (general population and healthy controls), (2) IBD, (3) primary biliary cholangitis (PBC; another cholestatic liver disease), (4) other liver related conditions (e.g. autoimmune hepatitis), and (5) other chronic health conditions (e.g. chronic fatigue syndrome). The number of PSC participants recruited to individual studies was mostly small and ranged from 13 to 341 (median = 65), with 13/17 studies including samples of less than 100 participants. Where reported, the mean age of PSC participants ranged from 35 to 53 years (median of means = 45), 51 to 81% were male (median = 68%), and the proportion of participants with co-occurring IBD ranged from 60 to 100% (median = 77%).

**Table 2:** Study characteristics for Review Question A

Study ID Country Quality◊	Study design	Sample size % Male	Age (mean) % IBD	PSC severity/stage	Recruitment setting	Comparator group(s)	Outcome measure(s)
Åberg 2012 Finland **	Cross-sectional	56 64%	53 NR	NR (all post-transplant)	All liver transplant patients transplanted in Finland between 1982 and May 2007	(1) Primary biliary cholangitis (2) Acute liver failure (3) Alcoholic cirrhosis (4) Liver tumour (5) Miscellaneous chronic liver disease group	(1) 15D instrument†
Ananthakrishnan 2010 USA **	Case–control	26 81%	41 100%	NR	Database from a tertiary IBD referral centre	(1) Inflammatory bowel disease††	(1) Short inflammatory bowel disease questionnaire†
Benito de Valle 2012 Sweden, UK *	Cross-sectional	182 70%	50 79%	Cirrhosis = 8% Decompensated liver disease = 6%	Computerised discharge diagnosis register of all hospitals in one region (Sweden); all patients at the outpatient clinic of one hospital (UK)	(1) General population††	(1) Medical outcomes study 36-item short form survey† (2) Chronic liver disease questionnaire† (2) Fatigue impact scale (3) Hospital anxiety and depression scale
Björnsson 2004 Sweden, UK **	Cross-sectional	93 65%	48 80%	Cirrhosis: 5% Ludwig’s fibrosis score: Stage 1 = 44%, Stage 2 = 21%, Stage 3 = 30%	All patients at one outpatient clinic in the UK and one outpatient clinic in Sweden	(1) General population†† (2) Inflammatory bowel disease	(1) Psychological wellbeing index (2) Beck depression inventory (3) Fatigue impact scale (4) Gastrointestinal symptoms rating scale
Cheung 2016 Canada **	Cross-sectional	99 51%	46 75%	Cirrhosis = 48% Decompensated liver disease = 16% Mean ALP: 243 U/L	Tertiary liver clinic in Canada (Toronto Centre for liver disease)	(1) Healthy controls (2) Inflammatory bowel disease (3) Primary biliary cholangitis (4) Non-autoimmune cholestatic liver disease	(1) Medical outcomes study 36-item short form survey† (2) Short inflammatory bowel disease questionnaire† (3) Liver disease quality of life questionnaire† (4) Patient health questionnaire-9 (depression)
Dyson 2015 UK **	Cross-sectional	40 78%	51 60%	Mean ALP: 275 U/L	All PSC patients under active follow-up at a regional liver centre in the UK	(1) Community controls (2) Inflammatory bowel disease†† (3) Primary biliary cholangitis††	(1) Fatigue impact scale (2) Epworth sleepiness scale (3) Hospital anxiety and depression scale (4) Composite autonomic symptom scale
Gorgun 2005 USA ***	Case–control	65 69%	43 100%	NR	Cleveland institutional review board-approved pelvic pouch database	(1) Inflammatory bowel disease	(1) Cleveland global quality of life questionnaire†
Haapamäki 2015 Finland **	Cross-sectional	341 54%	43 70%	Asymptomatic = 45% ERC-score mean (SD): 5.9 (3.4)	Tertiary referral centre (The Endoscopy Unit of Helsinki University Central Hospital)	(1) General population†† (2) Inflammatory bowel disease††	(1) 15D instrument†
Jones 2009 UK *	Cross-sectional	73 NR	NR NR	NR	The Freeman Liver Unit, Newcastle Hospital	(1) Healthy controls (2) Primary biliary cholangitis (3) Chronic fatigue syndrome (4) Non-alcoholic fatty liver disease (5) Vasovagal syncope	(1) Fatigue impact scale
Longworth 2003 UK **	Cohort	70 69%	50‡ NR	NR (all listed for transplant)	Consecutive cohorts of patients listed for liver transplant at six UK centres	(1) General population††	(1) EuroQol EQ-5D instrument†
Raszeja-Wyszomirska 2015b Poland *	Cross-sectional	102 72%	36 72%	Cirrhosis = 33%	A medical institution in Warsaw, Poland	(1) Healthy controls††	(1) Medical Outcomes Study 36-Item Short Form Survey† (2) PBC-40† (3) PBC-27†
Tarter 1991 USA *	Cross-sectional	52 42%¥	41¥ NR	Child–Pugh class: A = 49%, B = 38%, C = 14%	Presbyterian University Hospital of the University of Pittsburgh Health Sciences Center	(1) Laennec’s cirrhosis (2) Primary biliary cholangitis (3) Chronic active hepatitis (4) Hepatitis B (5) Hepatitis C (6) Cryptogenic cirrhosis (7) Alpha-1 antitrypsin deficiency	(1) Sickness impact profile†
Tillman 2011 Germany *	Cross-sectional	13 77%	42 NR	NR	Tertiary liver referral centre (Hannover Medical School)	(1) Primary biliary cholangitis (2) Hepatitis B (3) Hepatitis C (4) Autoimmune hepatitis (5) Other liver conditions	(1) Medical outcomes study 36-item short form survey† (2) Fatigue impact scale
Vannas 2020 Finland **	Cohort	48 67%	45 NR	Symptomatic patients (n = 32): Median MELD score = 13 Asymptomatic patients (n = 16): Median MELD score = 7	Transplantation and Liver Surgery Clinic (Helsinki University Hospital) and PSC follow-up registry (Helsinki University)	(1) General population††	(1) 15D instrument†
van Os 2007 Netherlands *	Cross-sectional	37 65%	44 NR	Cirrhosis = 14%, mean ALP = 258 U/L	Outpatient liver unit of the Erasmus Medical Centre, Rotterdam	(1) Primary biliary cholangitis	(1) Beck depression inventory
Wunsch 2016 Poland **	Cross-sectional	115 65%	35 NR	Mean ALP = 299 U/L Child–Pugh class: A = 53%, B = 40%, C = 7%	Liver Unit (Pomeranian Medical University) and the Liver and Internal Medicine Unit (Medical University of Warsaw)	(1) Primary biliary cholangitis	(1) Medical outcomes study 36-item short form survey† (2) PBC-40† (3) PBC-27†
Younossi 2000 USA *	Cross-sectional	29 27%¥	55¥ NR	No cirrhosis = 36%¥ Child–Pugh class¥: A = 35%, B = 22%, C = 2%	NR	(1) Healthy controls (2) Primary biliary cholangitis (3) Chronic obstructive pulmonary disease (4) Congestive heart failure (5) Type II diabetes,	(1) Medical outcomes study 36-item short form survey† (2) Chronic liver disease questionnaire†

*IBD* inflammatory bowel disease, *MELD* Mayo end-stage liver disease score, *NR* not reported, *ALP* alkaline phosphatase

◊Low quality = *, Moderate quality = **, High quality = ***. †Multi-dimensional quality of life questionnaire. ††Comparator group age- and gender-matched to PSC group. ¥ Value based on whole sample (including other disease groups). ‡ Median value

#### Quality assessment

For the two case–control studies, we rated one as high quality [34], and one as moderate quality [35] due to significant differences in permanent work disability between groups, which may have confounded findings. We rated two cohort studies as moderate quality due to missing QoL outcome data at follow-up: in one study < 80% of postal EQ-5D data returned [36] and in one study < 80% of 15-D data were returned or complete [37]. For the cross-sectional studies, we rated one study as high quality [38], six as moderate quality [11, 12, 39–42], and six as low quality [32, 43–47]. Low ratings were mainly due to a lack of clarity regarding the sample representativeness (e.g. sampling strategy not reported), significant differences between responders and non-responders, and due to the fact that analyses reported in the methods sections were missing from the results sections.

#### Evidence synthesis

Comparisons with healthy and community controls consistently indicated worse outcomes for people with PSC for: gastrointestinal symptoms [39], autonomic symptoms [41], physical and mental health functioning [11, 45, 46], and the impact of fatigue [41, 43]. There was mixed evidence for the comparison of QoL outcomes between people with PSC and the general population. Three studies reported no significant differences between these groups for QoL [12], fatigue [32], psychological well-being [32], depression [32, 39], or anxiety [32]. In contrast, one study suggested poorer mental health functioning for people with PSC [32], and one study found poorer QoL for people with PSC listed for a liver transplant compared with UK population norm values [36]. Another study compared people with PSC on the liver transplant list, who were symptomatic or asymptomatic (but indicated for liver transplant due to suspicious premalignant findings), with the general population in both the pre-transplant and post-transplant phase [37]. The symptomatic group had significantly poorer QoL compared with the general population, both pre-transplant and post-transplant. For the asymptomatic pre-malignant group, no significant difference was found at either timepoint [37]. Unexpectedly, one study found significantly higher levels of fatigue in the general population compared with people with PSC [39]. It should be noted, however, that the PSC sample in this study was small (n = 93) and that the response rate in the general population group was low (44%) which may have biased findings [39].

There was mixed evidence for the comparison of QoL between people with PSC and people with IBD only. One study reported significantly poorer QoL for people with IBD alone [12]. However, 45% of PSC participants in this study were asymptomatic. Two studies suggested no significant difference in QoL between people with PSC and IBD and people with IBD alone [34, 35]. However, one of these studies [35] assessed QoL with an IBD-specific measure, which is unlikely to capture PSC specific experiences. Two studies suggested no significant difference between PSC and IBD participants for the impact of fatigue [39, 41]. One study indicated no significant difference for psychological well-being [39], depression [39], or gastro-intestinal symptoms [39]. When compared with participants with PBC, PSC participants generally had higher QoL scores, but these differences were rarely significant. Seven studies indicated no significant difference between people with PSC and PBC for QoL [38, 44, 45, 48], fatigue [41, 43], or depression [40]. One study found significantly higher levels of daytime somnolence and autonomic symptoms in age- and gender-matched participants with PBC [41], however, the PSC group was small (n = 40). One study found that PSC participants had significantly greater QoL than participants with PBC, however, the PBC group were significantly older (57 vs. 35 years) and had a significantly higher proportion of participants with cirrhosis (46% vs. 26%) which may have biased the findings [47].

Studies comparing people with PSC with people with other liver-related conditions found generally similar levels of QoL [38, 48], physical functioning [44] and fatigue [44]. One study however, indicated significantly better mental health functioning in patients with PSC compared to those with hepatitis C [44]. One study comparing people with PSC with people with chronic obstructive pulmonary disorder (COPD), heart disease, and type II diabetes found that people with PSC had significantly greater physical health functioning [45], but their mental health functioning was significantly poorer [45]. Another study reported less severe fatigue in PSC participants compared with people with chronic fatigue syndrome, and more severe fatigue in PSC participants compared with people with vasovagal syncope [43]. However, the authors did not conduct any statistical analyses for these differences.

An initial sensitivity analysis was conducted restricting the evidence to studies judged as being moderate to high quality, however this did not explain the heterogeneity of findings. A further sensitivity analysis was conducted to explore whether any of the contradictory findings for the between-groups comparisons could be explained by differences in the age and gender of PSC participants compared with other groups. Eight of the 17 included studies age- and gender-matched PSC participants to the included comparator groups [12, 32, 35–37, 39, 41, 46], however, the evidence was still mixed when limited to these studies.

In summary, the evidence from this review for Question A suggests that people with PSC have poorer QoL than healthy controls, and generally similar QoL to people with other chronic conditions. When compared with the general population the evidence was mixed; three studies suggested no differences between groups for QoL, fatigue and mental health outcomes, however, one study found poorer mental health functioning in PSC [32] and one study unexpectedly found more severe fatigue in the general population[39].

### Question B: What factors are associated with impaired QoL in people with PSC?

#### Study characteristics

Ten studies met the inclusion criteria: nine were cross-sectional studies and one was a prospective cohort study, although only cross-sectional data were used in this review (Table 3). Factors associated with QoL comprised: demographic variables, symptoms, co-morbid conditions, clinical features of PSC (e.g. presence of liver cirrhosis), prognostic scoring systems (e.g. Mayo Risk Score [49]), and biochemical and genetic markers. Sample sizes ranged from 29 to 341 participants (median = 107), four of which had sample sizes < 100 participants. Where reported, the mean age of participants ranged from 35 to 55 years (median of means = 43), the proportion of men ranged from 54 to 72% (median = 67%), and the proportion of PSC participants with co-occurring IBD ranged from 61 to 79% (median = 71%).

**Table 3:** Study characteristics for Review Question B

Study ID Country Quality◊	Study design	Sample size % Male	Age (mean) % IBD	PSC severity/stage	Recruitment setting	Factor(s) correlated with QoL?	Outcome measure(s)
Benito de Valle 2012 Sweden, UK *	Cross-sectional	182 70%	50 79%	Cirrhosis = 8% Decompensated liver disease = 6%	Computerised discharge diagnosis register of all hospitals in one region (Sweden); all patients at the outpatient clinic of one hospital (UK)	(1) Age, (2) small/large duct PSC, (3) comorbid illness, (4) serum ALP	(1) Medical outcomes study 36-item short form survey† (2) Chronic liver disease questionnaire† (2) Fatigue impact scale (3) Hospital anxiety and depression scale
Cheung 2016 Canada **	Cross-sectional	99 51%	46 75%	Cirrhosis = 48% Decompensated liver disease = 16% Mean ALP: 243 U/L	Tertiary liver clinic in Canada (Toronto Centre for Liver Disease)	(1) Age, (2) gender, (3) marital status, (4) employment status, (5) symptoms, (6) history of decompensation	(1) Medical outcomes study 36-item short form survey† (2) Short inflammatory bowel disease questionnaire† (3) Liver disease quality of life questionnaire† (4) Patient health questionnaire-9 (depression)
Gotthardt 2014 Germany *	Cross-sectional	113 72%	44 63%	Mayo Risk score: low = 42%, intermediate = 22%, high = 4%. Data not reported for 35 participants	Tertiary care centre (University Hospital of Heidelberg)	(1) Age, (2) gender, (3) IBD status, (4) dominant stenosis, (5) Mayo risk score, (6) itch	(1) Medical outcomes study 36-item short form survey†
Haapamäki 2015 Finland **	Cross-sectional	341 54%	43 70%	Asymptomatic = 45% ERC-score mean (SD): 5.9 (3.4)	Tertiary referral centre (The Endoscopy Unit of Helsinki University Central Hospital)	(1) Age, (2) gender, (3) symptoms	(1) 15D instrument†
Kempinska-Podhorodecka 2017 Poland **	Cross-sectional	267 66%	55‡ NR	Median ALP = 354 U/L	Two medical centres in Poland	(1) Vitamin D receptor polymorphisms	(1) Medical outcomes study 36-item short form survey† (2) PBC-40† (3) PBC-27†
Raszeja-Wyszomirska 2015a Poland *	Cross-sectional	33 67%	35 61%	Cirrhosis = 7%, Mean ALP = 239 U/L	A single tertiary centre	(1) Fragility fractures	(1) Medical outcomes study 36-item short form survey† (2) PBC-40† (3) PBC-27†
Raszeja-Wyszomirska 2015b Poland *	Cross-sectional	102 72%	36 72%	Cirrhosis = 33%	A medical institution in Warsaw, Poland	(1) Age, (2) gender, (c) cirrhosis, (d) IBD status	(1) Medical outcomes study 36-item short form survey† (2) PBC-40† (3) PBC-27†
Tarter 1991 USA *	Cross-sectional	52 42%¥	41¥ NR	Child–Pugh class: A = 49%, B = 38%, C = 14%	Presbyterian University Hospital of the University of Pittsburgh Health Sciences Center	(1) Child’s-Pugh class	(1) Sickness Impact Profile†
Wunsch 2016 Poland **	Cross-sectional	115 65%	35 NR	Mean ALP = 299 U/L Child–Pugh class: A = 53%, B = 40%, C = 7%	Liver Unit (Pomeranian Medical University) and the Liver and Internal Medicine Unit (Medical University of Warsaw)	(1) Serum autotaxin	(1) Medical outcomes study 36-item short form survey† (2) PBC-40† (3) PBC-27†
Younossi 2000 USA *	Cross-sectional	29 27%¥	55¥ NR	No cirrhosis = 36%¥ Child–Pugh class¥: A = 35%, B = 22%, C = 2%	NR	(1) Age, (2) bilirubin, (3) Child’s-Pugh class	(1) Medical outcomes study 36-item short form survey† (2) Chronic liver disease questionnaire†

*IBD* inflammatory bowel disease, *NR* not reported, *ALP* alkaline phosphatase

◊Low quality = *, Moderate quality = **, High quality = ***. †Multi-dimensional quality of life questionnaire. ¥Value based on whole sample (including other disease groups). ‡ Median value

#### Quality assessment

Quality appraisal with the AXIS tool indicated four studies as being of moderate quality [11, 12, 42, 47] and six as being of low quality [32, 45, 46, 48, 50, 51]. Low ratings were mainly due to a lack of information about the selection of participants, the representativeness of the sample, a lack of information about non-responders, a high proportion of non-responders, and significant differences between responders and non-responders which may have biased outcomes.

#### Evidence synthesis

There was mixed evidence for the association between age, gender and QoL. Three studies suggested that older age was associated with poorer QoL [12, 32, 46], however two studies found no significant association [11, 51]. Two studies suggested women with PSC had significantly poorer mental health functioning [46], physical functioning [51] and limitations on routine activities due to emotional problems [51]. However, three studies indicated no association between gender and physical functioning [11], mental health functioning [11], or overall QoL [12]. One study found a positive association between employment and QoL, and a negative association for marital status [11].

With regards to symptoms, three studies indicated that the experience of itch [11, 12, 51], pain [12] and jaundice [12] were associated with worse QoL. For fatigue, however, there were contradictory findings: one study suggested a negative association with QoL [12] and one study indicated no significant association [11]. In each of these studies, fatigue was measured in different ways: one study [12] used a single item to assess the presence or absence of fatigue, whereas the other study [11] used the fatigue sub-scale of the PBC-40 [52]. Fragility fractures [50] and the number and severity of co-morbid conditions (of any kind) [32] were both found to be significantly associated with poorer QoL outcomes. Three studies did not find that the presence of co-morbid IBD impacted QoL [12, 46, 51]. However, another study found that people with PSC with more severe IBD had significantly poorer mental health functioning than those with milder IBD [11].

Two studies found that liver cirrhosis was associated with poorer physical functioning [32, 46], but not with mental health functioning [32, 46], and that people with large-duct PSC as opposed to small-duct PSC had poorer mental health functioning [32]. In contrast, another study indicated that having a dominant stricture was not associated with impaired QoL [51]. Three studies explored the impact of elevated serum alkaline phosphatase (ALP) on QoL outcomes [11, 32, 46], however only one of these studies found significantly worse QoL for people with elevated ALP [32]. One study further explored the impact of elevated alanine transaminase, gamma-glutamyl transferase and bilirubin on QoL outcomes, but only found a significant association between elevated bilirubin and one of eight subscales of the medical outcomes study short form (SF-36) (bodily pain) [46]. Four studies stratified disease severity with three prognostic scoring systems: the Child’s-Pugh score [53], the Mayo Risk score [49], and the modified ERC score [54]. Three studies found no association between QoL and disease severity as stratified with the Child’s-Pugh score [45, 48] or the Mayo Risk score [51]. One study unexpectedly found better QoL among participants with more advanced disease according to the ERC score, although the correlation was weak (β = 0.014; *p* = 0.045) [12]. One study suggested that people with a genetic polymorphism of the vitamin D receptor had poorer QoL [42]. One further study indicated no association between elevated serum autotaxin and QoL outcomes [47], except for the itch sub-domain of the PBC-40 and PBC-27 tools.

In summary the evidence from this review for Question B suggested that symptoms (e.g. itch and pain), co-morbid conditions, liver cirrhosis and large-duct PSC were associated with impaired QoL. In contrast, prognostic scoring systems and markers of disease progression commonly used as outcomes in clinical trials (e.g. ALP) did not consistently correlate with QoL.

### Question C: Which interventions are effective in improving QoL in people with PSC?

#### Study characteristics

Nine studies met the eligibility criteria: eight RCTs and a retrospective case note review (Table 4). Of the RCTs, seven investigated the efficacy of pharmacological interventions: two types of bile acids (ursodeoxycholic acid and nor-ursodeoxycholic acid), an immunosuppressant drug (infliximab), an engineered version of the hormone FGF19 (aldafermin or NGM282), antibiotics (vancomycin and metronidazole), and an antidepressant (fluoxetine). One RCT compared the efficacy of stent dilation with balloon dilation for PSC patients with dominant strictures [55]. The retrospective case note review assessed outcomes in people with PSC and ulcerative colitis who had undergone a restorative proctocolectomy with ileal pouch anal anastomosis compared with those who had not had surgery [56]. Sample sizes ranged from ten to 219 (median = 40). The majority of participants were men (median = 68%), in their early forties (median = 43), and with a diagnosis of IBD (median = 75%). For the RCTs, the length of study follow-up ranged from 10 to 260 weeks.

**Table 4:** Study characteristics for Review Question C

Study ID Study design	Country	Sample size Gender (%male)	Age (mean) % IBD	Disease stage/severity	Intervention group Comparator group	Follow-up (weeks)	Outcome measure(s)
Fickert 2017 RCT	Austria, Belgium, Denmark, Finland, Germany, UK Hungary, Spain Lithuania, Norway Netherlands, Sweden	161 68%	42 64%	Mean ALP = 445 U/L	norUrsodeoxycholic acid (0.5 g/1 g/1.5 g) Placebo	16	(1) Short health scale†
Hommes 2008 RCT	Netherlands	10 20%	45 NR	Mean ALP = 526 U/L	Infliximab Placebo	52	(1) Medical outcomes study 36-item short form survey†
Mayo 2019 RCT	France, Netherlands, USA, UK	62 61%	43 66%	Mean ALP = 364 U/L	Aldafermin (1 mg/3 mg) Placebo	62	(1) 5-D itch scale (2) Numerical rating scale for itch
Olsson 2005 RCT	Sweden, Norway, Denmark	219 70%	43 85%	Mean ALP = 729 U/L Asymptomatic = 45%	Ursodeoxycholic acid Placebo	260	(1) Medical outcomes study 36-item short form survey†
Ponsioen 2018 RCT	Belgium, France, Finland, Italy, Netherlands, Norway, Sweden, UK	65 69%	40 78%	Mean ALP = 306 U/L	Balloon dilation Stent dilation	104◊	(1) Medical outcomes study 36-item short form survey† (2) Amsterdam cholestatic complaints score
Rahimpour 2016 RCT	Iran	29 59%	36 75%	Mean ALP = 1015 U/L PSC Mayo Risk score = − 0.15	Vancomycin Placebo	12	(1) Fatigue impact scale (2) Visual analogue scale for itch
Tabibian 2013 RCT	USA	35 60%	40 71%	Mean ALP = 383 U/L PSC Mayo Risk score = 0.01	Vancomycin (125 mg/250 mg) Metronizadole (50 mg/500 mg)	12	(1) Fatigue impact scale (2) Visual analogue scale for itch
ter Borg 2004 RCT	Netherlands	11 82%	47 NR	Mean ALP = 164 U/L*	Fluoxetine Placebo	10	(1) Medical outcomes study 36-item short form survey† (2) Fatigue impact scale (3) Multidimensional fatigue inventory (4) Visual analogue scale for fatigue (5) Visual analogue scale for itch
Pavlides 2014 Retrospective cohort	UK	40 78%	NR 100%	NR	Restorative proctocolectomy with ileal pouch anal anastomosis No surgery	574	(1) Medical outcomes study 36-item short form survey† (2) Female sexual satisfaction index (3) International index of erectile function

*NR* not reported

^†^Multi-dimensional quality of life questionnaire. ††Mean of medians. ◊Quality of life outcomes reported at 12 weeks only. *Value for whole sample including 22 people with PBC

#### Quality assessment

For the RCT evidence there was a high risk of attrition bias in five trials [55, 57–60] due to high level or unequal drop-out, and a high or unclear risk of selective outcome reporting in seven trials [55, 57–62] as QoL outcomes were rarely listed in the available study protocols (Table 5). Two trials had a high risk of other bias, due to significant differences at baseline in the proportion of male participants [60, 63], significant differences in fatigue scores at baseline [60] and due to a lack of information about how continuous outcomes were dichotomised in the analysis [63]. The retrospective cohort study [56] was judged as having a moderate risk of bias as it is likely that patients with PSC who had surgery had more severe ulcerative colitis than patients who did not receive surgery, and this may have confounded the findings.

**Table 5:** Risk of bias assessment for the RCT evidence

	Sequence generation	Allocation concealment	Blinding of participants and personnel	Blinded outcome assessment	Incomplete outcome data	Selective outcome reporting	Other bias
ter Borg 2004	Low	Low	Low	Low	Low	Unclear	Low
Olsson 2005	Low	Low	Low	Unclear	High	Unclear	Low
Hommes 2008	Low	Unclear	Low	Unclear	High	Unclear	Low
Tabibian 2013	Low	Unclear	Low	Low	High	High	High
Rahimpour 2016	Low	Low	Low	Low	Low	Low	High
Fickert 2017	Low	Unclear	Low	Low	High	High	Low
Ponsioen 2018	Low	Unclear	High	Unclear	High	High	Low
Mayo 2019	Low	Low	Low	Low	Low	High	Low

#### Evidence synthesis

For the RCT evidence, five studies indicated no significant differences between intervention groups for QoL [55, 57, 59, 61], the impact of fatigue [59, 61], itch [61, 62] or cholestatic symptoms [55]. One study [63] reported significant within-group improvements for itch at 12 weeks’ follow-up for the vancomycin and placebo group, however, between-group differences were not reported. One study [60] also reported a significant within-group improvement for itch for participants at 12 weeks’ follow-up in the high-dose metronidazole group, but not for the other three intervention groups (between-groups differences were not conducted). No within-group improvements were found for fatigue in any of the four intervention groups. In one study [58], QoL data (measured with the Short Form-36) were only available for seven participants and were not analysed.

The retrospective cohort study found poorer QoL for patients who had undergone surgery for ulcerative colitis compared with those who had not had surgery, however, these differences were not significant [56]. There were no differences between groups for male sexual function. The authors were unable to assess female sexual function due to the small number of female participants (n = 7).

In summary, this review for Question C found no studies where interventions improved QoL, the impact of fatigue, or specific symptoms such as itch and pain.

## Discussion

### Main findings

Despite the broad scope of this review and our inclusion of studies assessing QoL as well as specific domains of QoL, we identified few studies for each review question, and we were unable to conduct any meta-analyses. Most of the identified studies for Question A compared QoL outcomes in people with PSC with other groups (n = 17). The Question B search found ten studies exploring whether specific factors or attributes were associated with impaired QoL. However, seven of these studies were also included in Question A. For Question C, only eight RCTs were found assessing the effect of pharmacological or surgical interventions on QoL, three of which studies focused only on patient-reported symptoms [60, 62, 63] as opposed to composite measures of QoL.

In Question A the evidence indicated poorer QoL for people with PSC compared with healthy controls, comparable QoL to people with other chronic conditions, but mixed findings for comparisons with the general population. The evidence in Question B suggested that symptoms, IBD severity, liver cirrhosis, and large-duct PSC were all associated with impaired QoL. No associations were found between QoL and PSC severity measured with surrogate markers of disease progression (e.g. ALP) or one of three prognostic scoring systems. In Question B many of the identified factors were explored in a single study which made it challenging to derive any firm conclusions. In Question C, no interventions were found to improve QoL outcomes. With the exception of five studies [12, 32, 42, 57, 59], in each review study sample sizes were small, with no more than 120 participants with PSC. Various QoL measures were used across studies, which may explain some of the heterogeneity of findings.

### Strengths and limitations

To our knowledge this is the first paper to systematically review the literature for studies exploring QoL in PSC. We conducted a comprehensive search across five electronic databases, as well as reviewing reference lists of relevant studies and conducting a forward citation search for included studies. Two reviewers (EM, AMK) independently screened 10% of identified references and all full-text papers, with a high-level of agreement. The review was limited in that data extraction and the quality of individual studies was assessed by a single reviewer. Due to resource limitations, we only included English language papers.

In contrast to previous literature reviews [24, 25], one of our inclusion criteria was studies where the majority of participants had a PSC diagnosis. This will have limited the available evidence, because a number of studies have explored QoL more broadly with people with cholestatic disease or chronic liver disease, which may include a sub-set of PSC participants. None of the studies we included had a mixed population, because no identified studies included a majority of PSC participants. We excluded 19 studies in which 3–36% of the sample had PSC, as well as eight studies which did not provide information about the diagnoses of participants. Authors were contacted to request this information and the disaggregated data, but these were only provided for a single study [61]. It is challenging to recruit participants with rare conditions such as PSC [15], however, this inclusion criterion is important because there are key differences between PSC and with other liver conditions. For example, unlike PSC, PBC predominantly affects women, is not associated with IBD, and has a more predictable clinical course [7, 64]. Factors such as these are likely to affect QoL, and so extrapolating findings from other liver-related conditions (where participants with PSC are in a minority) may not be appropriate.

### Implications and gaps in the literature

It is clear that PSC can have a detrimental impact on QoL. However, findings from the studies identified were mixed for comparisons of people with PSC with the general population, even though participants were age- and gender-matched. These study findings were based on scores from generic questionnaires, such as the SF-36 [65] and the 15-D instrument [66], and it is possible that these measures only have a limited relationship to the experiences of people with PSC. Six of these studies were also small scale: two included < 50 participants, and four included < 100 participants. This reduces our confidence in the findings, with a risk of type II errors (i.e. failing to reject the null hypothesis when it is false). Another possible hypothesis is that, at the group level, QoL for people with PSC is similar to the general population, due to the relatively high proportion of people with PSC who are asymptomatic. Only one study in this review reported on the proportion of asymptomatic participants, which was as high as 45% [12]. These data were missing from the other published reports, and so based on the available evidence this question remains unanswered.

A co-morbid diagnosis of IBD in people with PSC was not found to impact on QoL, however the severity of IBD symptoms was found to be independently associated with impaired QoL. Although IBD in PSC tends to be mild [67], this finding suggests it is important to monitor IBD related symptoms and impacts when assessing QoL in PSC [11]. As expected, the experience of symptoms such as itch, pain and jaundice were associated with worse QoL, but there were contradictory findings for the impact of fatigue. Fatigue is a debilitating symptom which is commonly experienced by people with PSC [68]. These findings suggest that generic measures of QoL may not adequately capture the impact of fatigue, indicating a need for a disease-specific measure of QoL for PSC.

Surrogate markers of disease progression are commonly used as primary outcomes in clinical trials in place of “harder” outcomes such as cirrhosis or mortality, which can take a long time to occur making them unsuitable for trial design [69]. Despite the common use of these markers, no single method has been recommended to predict individual patient prognosis in PSC [6]. In this review, disease progression as measured with surrogate markers (e.g. ALP) and with prognostic scoring systems (which include such markers) was not found to correlate with assessments of QoL. In light of these findings we recommend the inclusion of assessments of QoL in all future trials. We only identified five RCTs which used a composite measure of QoL as an outcome, which suggests this integration is currently lacking.

The evidence base was limited across all review questions with few studies included in each comparison, many of which had small samples. In addition, many studies reported QoL data narratively or only provided *p* values which meant it was not possible to explore the magnitude of significant findings. Key gaps in the literature include the impact of fatigue on QoL, only explored in two studies with conflicting findings, and the impact of IBD severity, only explored in a single study. The available literature only explored the association of clinical or demographic factors with QoL. Studies exploring psychological or social factors, such as self-efficacy or social support, which can also impact on QoL [70], were lacking. We found very limited evidence exploring QoL in people with large-duct PSC compared with small-duct PSC, as well as in people with and without a dominant stricture, features which are associated with the severity of PSC, transplant-free survival and risk of cancer [1, 2]. Staging or stratifying PSC is challenging, however, newer methods such as the enhanced liver fibrosis (ELF) test have shown greater sensitivity than existing models in predicting outcomes, particularly in the earlier stages of the condition [71, 72]. We did not find any evidence exploring how this method of staging correlates with the lived experiences of people with PSC. Future research studies should address this topic.

## Conclusion

This review found few studies exploring QoL for people with PSC. Those included found worse QoL for people with PSC than for healthy controls, and similar QoL to people with other chronic conditions. Comparisons with the general population were mixed. Many studies included small numbers of participants, which is due in large part to the rarity of the condition. Those studies found that symptoms, severity of IBD, liver cirrhosis, and large-duct PSC were all associated with worse QoL. No interventions indicated any evidence of benefit on QoL. Studies assessing disease severity using three prognostic scoring systems and markers of disease progression did not find any correlations with QoL. We recommend that larger-scale clinical research studies are conducted, measuring QoL alongside clinical and laboratory-based outcomes. We also recommend the development of a valid, responsive, and PSC-specific measure of QoL for use in such studies, since generic measures of QoL may not cover all issues of importance to people with PSC, such as fatigue.

## Supplementary Information

**Additional file 1.** Full MEDLINE search strategy.

## Data Availability

The datasets during and/or analysed during the current study available from the corresponding author on reasonable request.
