# Supplementary material for: Quality of life in primary sclerosing cholangitis: a systematic review
Source: Health Qual Life Outcomes. 2021 Mar 20;19:100. doi: 10.1186/s12955-021-01739-3 (PMC7981996; doi:10.1186/s12955-021-01739-3)
Supplement: Supplementary file 1 — Additional file 1. Full MEDLINE search strategy. [file 12955_2021_1739_MOESM1_ESM.docx]

## Additional File - OVID Medline Strategy

| **Ovid MEDLINE Search Strategy** | |
| --- | --- |
| 1 | Cholangitis, Sclerosing/ |
| 2 | primary sclerosing cholangitis.mp. |
| 3 | cholestasis/ or cholestasis, extrahepatic/ or cholestasis, intrahepatic/ |
| 4 | cholestatic liver disease*.mp. |
| **5** | **1 or 2 or 3 or 4** |
| 6 | "Quality of Life"/px [Psychology] |
| 7 | (quality of life or life quality or QOL).mp. |
| 8 | (HQL or HRQOL or H QOL or HRQOL or HR QOL or HR QL or HRQL).mp. |
| 9 | (wellbeing or well being).mp. |
| 10 | Health Status/ |
| 11 | Psychometrics/ |
| 12 | "Surveys and Questionnaires"/ |
| 13 | (short form health survey or shortform health survey).mp. |
| 14 | (sf36 or sf 36 or short form 36 or shortform 36 or sf thirtysix or sf thirty six or shortform thirtysix or shortform thirty six or short form thirtysix or short form thirty six).mp. |
| 15 | (sf6 or sf 6 or short form 6 or shortform 6 or sf six or sfsix or shortform six or short form six).mp. |
| 16 | (sf12 or sf 12 or short form 12 or shortform 12 or sf twelve or sftwelve or shortform twelve or short form twelve).mp. |
| 17 | (sf16 or sf 16 or short form 16 or shortform 16 or sf sixteen or sfsixteen or shortform sixteen or short form sixteen).mp. |
| 18 | (sf20 or sf 20 or short form 20 or shortform 20 or sf twenty or sftwenty or shortform twenty or short form twenty).mp. |
| 19 | (euroqol* or euro qol* or eq5d* or eq 5d*).mp. |
| 20 | (health utilities index or hui or hui1 or hui2 or hui3).mp. |
| 21 | rosser.mp. |
| 22 | (quality of wellbeing* or quality of well being* or QWB*).mp. |
| 23 | (nottingham health profile or NHP).mp. |
| 24 | sickness impact profile/ |
| 25 | (sickness impact profile or SIP).mp. |
| 26 | (psychological general well being* or psychological general wellbeing* or PGWB*).mp. |
| 27 | (patient health questionnaire or PHQ-9 or PHQ9).mp. |
| 28 | (PBC-40 or PBC40 or PBC-27 or PBC27).mp. |
| 29 | (inflammatory bowel disease questionnaire or IBDQ or SIDBQ).mp. |
| 30 | (liver disease quality of life* or LDQOL).mp. |
| 31 | (chronic liver disease questionnaire or CLDQ).mp. |
| 32 | (fatigue impact scale or FIS).mp. |
| **33** | **6 or 7 or 8 or 9 or 10 or 11 or 12 or 13 or 14 or 15 or 16 or 17 or 18 or 19 or 20 or 21 or 22 or 23 or 24 or 25 or 26 or 27 or 28 or 29 or 30 or 31 or 32** |
| **34** | **5 and 33** |
